# Hepatitis C Virus Infection Suppresses the Interferon Response in the Liver of the Human Hepatocyte Chimeric Mouse

**DOI:** 10.1371/journal.pone.0023856

**Published:** 2011-08-23

**Authors:** Masataka Tsuge, Yoshifumi Fujimoto, Nobuhiko Hiraga, Yizhou Zhang, Mayu Ohnishi, Tomohiko Kohno, Hiromi Abe, Daiki Miki, Michio Imamura, Shoichi Takahashi, Hidenori Ochi, C. Nelson Hayes, Fuyuki Miya, Tatsuhiko Tsunoda, Kazuaki Chayama

**Affiliations:** 1 Division of Frontier Medical Science, Department of Medicine and Molecular Science, Programs for Biomedical Research, Graduate School of Biomedical Sciences, Hiroshima University, Hiroshima, Japan; 2 Natural Science Center for Basic Research and Development, Hiroshima University, Hiroshima, Japan; 3 Liver Research Project Center, Hiroshima University, Hiroshima, Japan; 4 Third Department of Internal Medicine, Hiroshima General Hospital, Hatsukaichi, Japan; 5 Laboratory for Liver Diseases, SNP Research Center, The Institute of Physical and Chemical Research (RIKEN), Hiroshima, Japan; 6 Laboratory for Medical Informatics, SNP Research Center, The Institute of Physical and Chemical Research (RIKEN), Yokohama, Japan; Pohang University of Science and Technology, Korea

## Abstract

**Background and Aims:**

Recent studies indicate that hepatitis C virus (HCV) can modulate the expression of various genes including those involved in interferon signaling, and up-regulation of interferon-stimulated genes by HCV was reported to be strongly associated with treatment outcome. To expand our understanding of the molecular mechanism underlying treatment resistance, we analyzed the direct effects of interferon and/or HCV infection under immunodeficient conditions using cDNA microarray analysis of human hepatocyte chimeric mice.

**Methods:**

Human serum containing HCV genotype 1b was injected into human hepatocyte chimeric mice. IFN-α was administered 8 weeks after inoculation, and 6 hours later human hepatocytes in the mouse livers were collected for microarray analysis.

**Results:**

HCV infection induced a more than 3-fold change in the expression of 181 genes, especially genes related to Organismal Injury and Abnormalities, such as fibrosis or injury of the liver (P = 5.90E-16 ∼ 3.66E-03). IFN administration induced more than 3-fold up-regulation in the expression of 152 genes. Marked induction was observed in the anti-fibrotic chemokines such as *CXCL9*, suggesting that IFN treatment might lead not only to HCV eradication but also prevention and repair of liver fibrosis. HCV infection appeared to suppress interferon signaling via significant reduction in interferon-induced gene expression in several genes of the IFN signaling pathway, including *Mx1*, *STAT1*, and several members of the *CXCL* and *IFI* families (P = 6.0E-12). Genes associated with Antimicrobial Response and Inflammatory Response were also significantly repressed (P = 5.22×10^−10^ ∼ 1.95×10^−2^).

**Conclusions:**

These results provide molecular insights into possible mechanisms used by HCV to evade innate immune responses, as well as novel therapeutic targets and a potential new indication for interferon therapy.

## Introduction

Chronic hepatitis C virus (HCV) infection is one of the most serious global health threats, affecting more than 170 million people worldwide [1]–[3]. Interferon is administered to chronic hepatitis C patients to attempt to eradicate the virus and to prevent the development of advanced liver diseases such as chronic hepatitis, cirrhosis, and hepatocellular carcinoma (HCC), with limited success. While the overall eradication rate of HCV has improved since the introduction of pegylated-interferon (PEG-IFN) and ribavirin (RBV) combination therapy, the sustained viral response (SVR) rate of genotype 1b with high viral load still remains only 40–50% [4]–[6]. Viral and host factors, such as HCV RNA titer, viral substitutions in HCV core or NS5A region, age, gender, liver fibrosis, and SNPs in *IL-28B locus*, are significantly associated with the effects of PEG-IFN and RBV combination therapy [7]–[15], but the precise molecular mechanisms remained unclear.

Recently, some HCV-related structural as well as non-structural proteins have been reported to be associated with host proteins and affect innate immunity or lipid metabolism. RIG-I (retinoic acid inducible gene I) and Mda5 (melanoma differentiation-associated gene 5) are known to activate the type I interferon signaling pathway by interacting with adaptor protein IPS-1/MAVS/VISA/Cardif [16]–[18]. In the presence of HCV infection, the viral non-structural protein NS3/4A, which has serine protease activity, can cleave and inactivate IPS-1 [19]. TLR (Toll like receptor) is a sensor of RNA or DNA and is known to play various roles in viral infection. Abe et al. demonstrated that HCV non-structural protein NS5A inhibits the recruitment of interleukin-1 receptor-associated kinase 1 by interacting with MyD88 and impairs cytokine production in response to TLR ligands [20]. HCV core protein is also known to interact with host proteins. The core protein promotes hepatic steatosis, insulin resistance and hepatocarcinogenesis through activation of host proteins such as PPARα and MAPK [21]–[26]. However, these reports were based on *in vitro* analysis of cell lines or used human liver tissues in which results were complicated by adaptive immune responses, and it has been difficult to evaluate the direct impact of HCV infection and interferon administration on human hepatocytes.

Mercer and colleagues developed a human hepatocyte chimeric mouse [27] derived from the severely immunocompromised SCID mouse, in which mouse liver cells were extensively replaced with human hepatocytes [27], [28]. This mouse model facilitates continuous HCV infection and makes it possible to analyze the effects of drugs and viral infection on human hepatocytes under immunodeficient conditions [29], [30]. To analyze the putative effects of HCV infection or IFN administration without the adaptive immune response, we constructed an HCV carrier mouse model using the human hepatocyte chimeric mouse and performed cDNA microarray analysis using human hepatocytes dissected from the mouse livers. The results are intended to reflect the direct impacts of HCV infection and IFN administration on human hepatocytes and may help in elucidating HCV immune evasion mechanisms.

## Materials and Methods

### Human Serum Samples

Serum samples were obtained from HCV carriers after obtaining written informed consent for the donation and evaluation of blood samples. Inocula contained high viral loads of genotype 1b HCV RNA (6.9 log copies/ml). The experimental protocol met the ethical guidelines of the 1975 Declaration of Helsinki and was approved by the Hiroshima University Ethical Committee.

### Human Hepatocyte Chimeric Mice Experiments

The uPA^+/+^/SCID^+/+^ mice and transplantation of human hepatocytes were performed as described previously [28]. All mice were transplanted with hepatocytes from the same donor. Human hepatocyte chimeric mice, in which liver cells were largely (>90%) replaced with human hepatocytes, were used to reduce potential influence by mouse-derived mRNA. The experiments were performed in accordance with the guidelines of the local committee for animal experiments at Hiroshima University.

**Figure 1:**
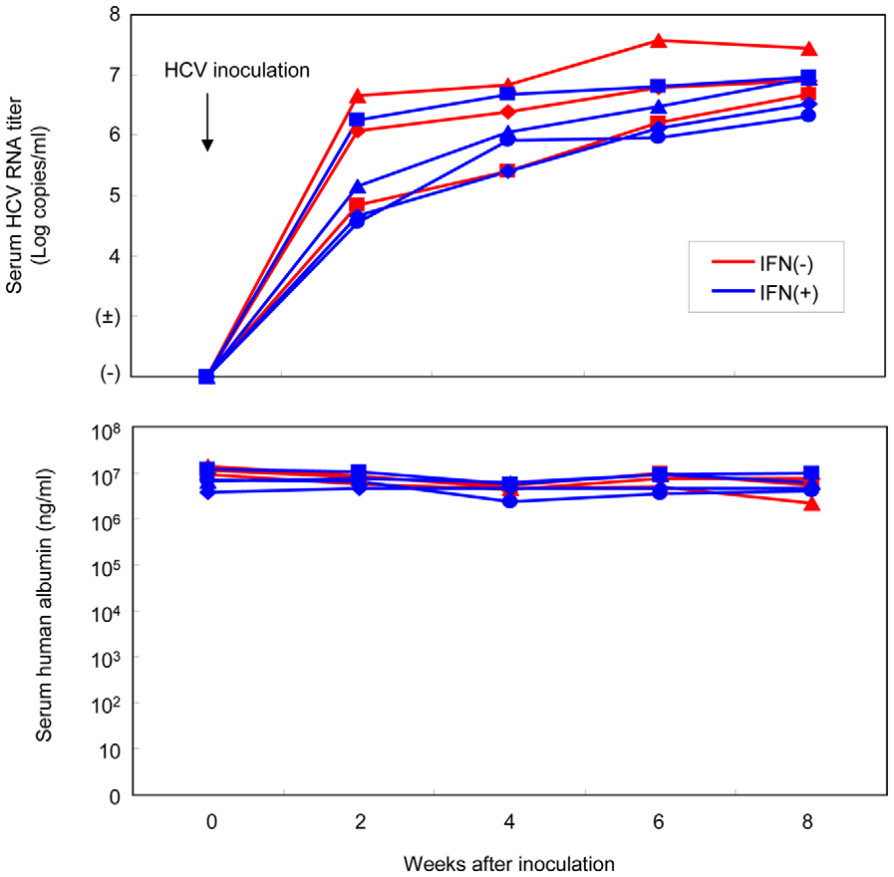
Change in HCV titers and human albumin levels in mouse serum. HCV RNA titers (upper panel) and human albumin levels (lower panel) in chimeric mouse sera after inoculation are shown. The horizontal axis indicates weeks after inoculation. Mouse sera were collected every two weeks after inoculation, and serum HCV RNA and human albumin levels were measured. Results were similar for all mice.

A total of 15 chimeric mice were prepared and assigned to four experimental groups. Group A contained four mice that were neither infected with HCV nor treated with IFN. Group B consisted of four uninfected mice that were administered IFN-α (7,000 IU/g body weight) 6 h before sacrifice. Groups C and D were inoculated via the mouse tail vein with human serum containing 4×10^5^ copies of HCV particles, and Group D was administered INF- α at the same time as Group B. After inoculation, we collected mouse sera every two weeks and analyzed serum HCV RNA levels by real time PCR. All seven mice developed measurable viremia 4 weeks after inoculation. The levels of the virus titer reached over 6 Log_10_ copies/ml 8 weeks after inoculation (Figure 1). Conversely, serum human albumin levels remained more than 2×10^6^ ng/ml in each mouse during 6 weeks after inoculation (Figure 1). Eight weeks after inoculation, when serum HCV RNA levels had plateaued, IFN-α (7,000 IU/g body weight) was administered to the four mice in Group D as well as the four uninfected mice in Group B. Six hours after IFN administration all 15 mice were sacrificed. Infection, extraction of serum samples, and sacrifice were performed under ether anesthesia as described previously [29]–[31]. Human albumin levels in mouse serum were measured with a Human Albumin enzyme-linked immunosorbent assay (ELISA) Quantitation kit (Bethyl Laboratories Inc., Montgomery, TX) according to the instructions provided by the manufacturer. Serum samples obtained from mice were aliquoted and stored in liquid nitrogen until use.

### Analysis of HCV markers

For quantitative analysis of HCV RNA, 10 µl samples of mouse serum were used. Total RNA was extracted using Sepa Gene RV-R (Sanko Junyaku Co., Ltd., Tokyo, Japan) and dissolved with 8.8 µL of RNase free water and reverse transcribed (RT). RT reactions were performed with 20 µl of the reaction mixtures, containing random primer (Takara Bio Inc., Shiga, Japan), RT buffer and M-MLV reverse transcriptase (ReverTra Ace, TOYOBO Co., Osaka, Japan) according to the instructions provided by the manufacturer. After the RT reaction, HCV RNA was quantified by real-time PCR using the 7300 Real-Time PCR System (Applied Biosystems, Foster City, CA). Amplification was performed as described previously [29], [30]. The lower detection limit of this assay is 300 copies. For detection of small amounts of HCV RNA, we also performed nested PCR. Amplification conditions were as described previously [29], [30].

### Dissection of mouse livers and total RNA extraction from human heptocytes in the mouse livers

All 15 chimeric mice were sacrificed by anesthesia with diethyl ether. Human hepatocytes were finely dissected from mouse livers, submerged in RNA *later*® solution (Applied Biosystems), and stored in liquid nitrogen. Total RNA was extracted using the Qiagen RNeasy Mini Kit according to the manufacturer protocol (Qiagen Inc., Valencia, CA). RNA quality was assessed using ultraviolet absorption at 260 nm/280 nm (NanoDrop Technologies, Wilmington, DE) and agarose gel electrophoresis. Microarray analysis was performed using the Affymetrix GeneChip Human Gene U133Plus2.0 Array, which interrogates 38,500 genes across 54,675 distinct probes (Affymetrix, Santa Clara, CA). The Affymetrix GeneChip Whole Transcript Sense Target Labeling Assay Manual Version 4 was used for complementary DNA (cDNA) generation, hybridization, and array processing. Briefly, 300 ng of total RNA underwent first-strand and second-strand cDNA synthesis. Complementary RNA was generated and used to produce sense-strand cDNA, which was fragmented and end-labeled with biotin. Biotin-labeled cDNA was hybridized to the Human Gene 1.0 ST Array for 16 hours at 45°C using the GeneChip Hybridization Oven 640 (Affymetrix). Washing and staining with streptavidin-phycoerythrin was performed using the GeneChip Fluidics Station 450, and images were acquired using the Affymetrix Scanner 3000 (Affymetrix).

### Microarray Data Analysis and Hierarchical Clustering

Fluorescence intensities captured by the Affymetrix GeneChip Scanner were converted to numerical values using the Affymetrix GeneChip Operating Software, were log2 transformed, and were standardized using quantile normalization with the Robust Multiarray Analysis (RMA) algorithm [32], [33]; this method normalizes the distribution of probe intensities for all the gene arrays in a given set.

Obtained gene expression profiles were analyzed using GeneSpring GX 10.0.2 software (Tomy Digital Biology, Tokyo, Japan). Expression ratios were calculated and normalized per chip to the 50th percentile and finally normalized per gene to medians. We worked on a pre-screened list of 32,885 probes obtained after filtering the data for outliers, negative and positive controls, and on the quality flag Cy3 signals being “well above background.” To pass this last flag, Cy3 net signals needed to be positive and significant, with g(r)BGSubSignal greater than 2.6 g(r) BG_SD. To determine if there were genes differentially expressed among samples, we performed two Welch's t-tests (P<0.01) on this prescreened list of genes: one without correction and one with Benjamini and Hochberg's correction. Complete linkage hierarchical clustering analysis was applied using Euclidean distance, and differentially expressed genes were annotated using the information from the Gene Ontology Consortium. Global molecular networks and comparisons of canonical pathways were generated using Ingenuity™ Pathway Analysis 8.6 (Ingenuity™ Systems, CA, USA).

### Real time PCR for analyzing the mRNA expression in the human hepatocytes

Total RNA was extracted from the implanted human hepatocytes in the mouse livers using RNeasy Mini Kit (Qiagen) and reverse-transcribed using ReverTra Ace (TOYOBO, Osaka, Japan) with random primer in accordance with the instructions supplied by the manufacturer. The selected cDNA were quantified by real-time PCR using the 7300 Real-Time PCR System (Applied Biosystems, Foster City, CA), and the expression of GAPDH served as a control. Amplification was performed in a 25 µl reaction mixture containing 12.5 µl SYBR Green PCR Master Mix (Applied Biosystems), 5 pmol of forward primer, 5 pmol of reverse primer, and 1 µl of cDNA solution. After incubation for 2 min at 50°C, the sample was denatured for 10 min at 95°C, followed by a PCR cycling program consisting of 40 cycles of 15 s at 95°C, 30 s at 55°C, and 60 s at 60°C.

### Statistical analysis

Differences between groups were examined for statistical significance using the Student's *t*- test.

## Results

### Change of gene expression with HCV infection

**Figure 2:**
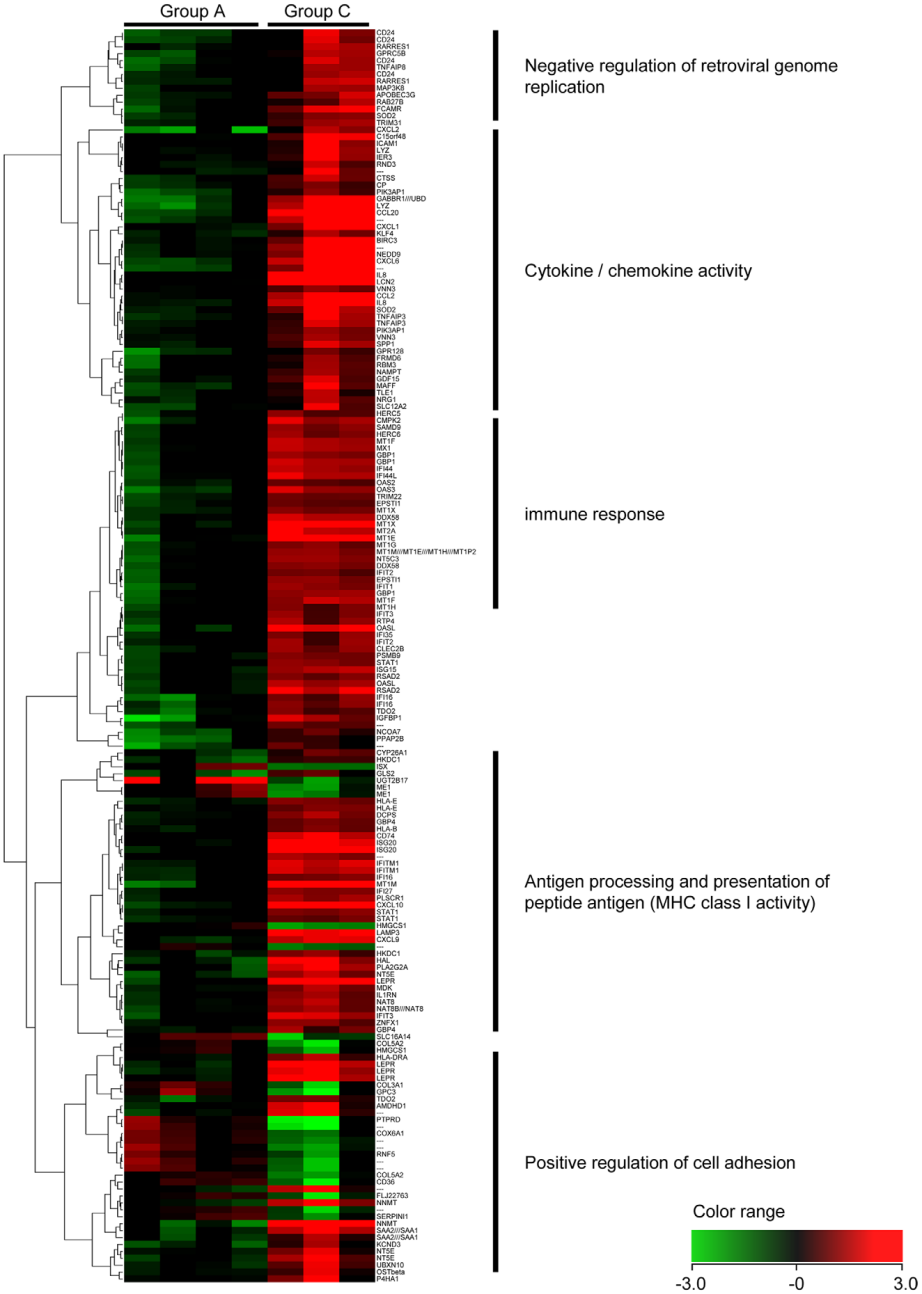
Hierarchical clustering analysis of 181 genes associated with HCV infection. To analyze the influence of HCV infection on human hepatocytes, clustering analysis on gene expression was performed between Group A (without HCV infection; 4 columns on the left side) and Group C (with HCV infection; 3 columns on the right side). 157 genes were up-regulated following HCV infection, including interferon-stimulated genes (ISGs) such as *MX1* and genes in the *CXCL* and *IFI* families, and 24 genes were down-regulated, including *ME1* and *HMGCS1*.

To analyze the effect of HCV infection on gene expression in human hepatocytes, we compared the gene expression profiles between Group A (without HCV infection) and Group C (with HCV infection). Among the 2,519 genes that remained significant after screening by Welch's t-test, more than 3.0-fold expression changes between groups were observed in 181 genes. 157 of these 181 genes were up-regulated following HCV infection, and the other 24 were down-regulated. Cluster analysis of the 181 genes is shown in Figure 2, and the top 20 up-/down-regulated genes by HCV infection are listed in Tables 1 and 2, respectively.

**Table 1:** The top 20 genes up-regulated with HCV infection.

Probe set	Unigene code	Gene symbol	Fold change	P value
202237_at	Hs.503911	NNMT	33.16	1.66E-03
205476_at	Hs.75498	CCL20	30.23	1.59E-04
202859_x_at	Hs.551925	IL8	30.16	4.42E-04
206336_at	Hs.164021	CXCL6	25.52	1.86E-03
217546_at	Hs.647370	MT1M	24.69	2.46E-04
212531_at	Hs.204238	LCN2	24.17	9.19E-04
209894_at	Hs.705413	LEPR	23.77	5.83E-04
204533_at	Hs.632586	CXCL10	23.61	1.47E-05
213797_at	Hs.17518	RSAD2	20.43	7.31E-05
204439_at	Hs.715563	IFI44L	17.92	9.73E-04
213975_s_at	Hs.706744	LYZ	15.22	1.10E-03
206643_at	Hs.190783	HAL	14.88	3.98E-03
216598_s_at	Hs.303649	CCL2	14.76	6.99E-03
235229_at	Hs.332649		13.93	6.22E-04
205890_s_at	Hs.714406	GABBR1///UBD	13.67	1.46E-03
33304_at	Hs.459265	ISG20	13.58	5.61E-05
205569_at	Hs.518448	LAMP3	10.96	3.58E-05
204470_at	Hs.789	CXCL1	10.90	9.06E-03
208607_s_at	Hs.632144	SAA1///SAA2	10.40	4.56E-03
205302_at	Hs.642938	IGFBP1	9.55	1.10E-02

**Table 2:** The top 20 genes down-regulated with HCV infection.

Probe set	Unigene code	Gene symbol	Fold change	P value
207245_at	Hs.575083	UGT2B17	20.04	2.15E-02
214043_at	Hs.446083	PTPRD	6.81	2.57E-02
214416_at	Hs.702961		6.36	3.51E-02
209220_at	Hs.713537	GPC3	5.40	4.40E-02
238029_s_at	Hs.504317	SLC16A14	4.90	1.16E-02
231594_at			4.59	1.87E-02
1556824_at	Hs.702604		4.40	2.89E-02
232707_at	Hs.567637	ISX	4.30	6.52E-03
205822_s_at	Hs.397729	HMGCS1	4.23	1.95E-04
204058_at	Hs.21160	ME1	4.06	2.15E-02
1555084_at			3.95	4.10E-02
215076_s_at	Hs.443625	COL3A1	3.92	2.99E-02
209555_s_at	Hs.120949	CD36	3.91	4.49E-02
221729_at	Hs.445827	COL5A2	3.89	2.60E-02
217676_at	Hs.696837		3.86	8.73E-03
233604_at	Hs.280892	FLJ22763	3.82	2.44E-02
1563298_at	Hs.352254		3.64	1.97E-02
224344_at	Hs.497118	COX6A1	3.34	1.68E-02
237031_at	Hs.146276		3.22	4.62E-04
216018_at	Hs.534342	RNF5	3.19	3.13E-02

**Figure 3:**
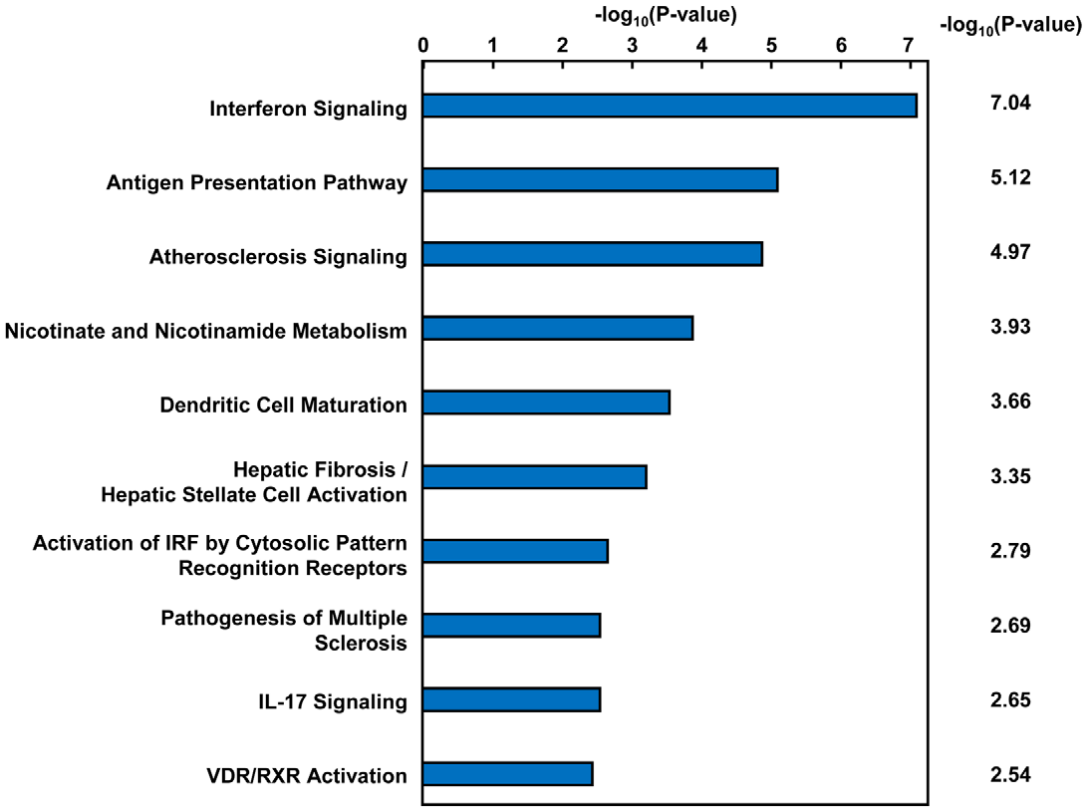
The effects of HCV infection on canonical pathways. To analyze the effects of HCV infection on canonical pathways, pathway analysis was performed using the 181 genes identified to be significantly up- or down-regulated following HCV infection. The IFN signaling pathway was the most significantly affected by HCV infection. Statistical analysis was performed using Fisher's exact test.

It is well known that chronic HCV infection triggers multiple biological responses. To analyze biological significance and regulatory pathways involved in the changes observed, we performed network analysis with the 181 genes using Ingenuity™ Pathway Analysis (IPA). As shown in Table 3, most of the 181 genes (e.g. *CXCL9*, *CXCL10*, *IFIT3* and *Mx1*, which are well known interferon-stimulated genes (ISGs)) belonged to categories such as Organismal Injury and Abnormalities, Inflammatory Response, and Cell-To-Cell Signaling and Interaction. Through canonical pathway analysis of the 181 genes using Ingenuity Pathways Analysis, 10 canonical pathways significantly affected by HCV infection were identified, with interferon signaling as the most significant (Figure 3). These results indicate that the intra-hepatic innate immune response was strongly activated by HCV infection in human hepatocytes.

**Table 3:** The effect of HCV infection on biological functions by category.

Category	P value	Up-regulated genes in network		Down-regulated genes in network	
		Number of genes	Representative genes	Number of genes	Representative genes
Organismal Injury and Abnormalities	5.90E-16–3.66E-03	27	CXCL1, CXCL6, CXCL9, CXCL10, IFIT1, IFIT3, MX1, etc.	1	SERPINI1
Cancer	1.81E-13–5.73E-03	54	BIRC3, CXCL9, CXCL10, GBP1, IFIT3, IGFBP1, ISG20, MAP3K8, etc.	4	CD36, COL3A1, GPC3, RNF5
Inflammatory Response	9.31E-13–5.89E-03	39	APOBEC3G, CCL2, CXCL9, CXCL10, IL8, MX1, STAT1, TRIM22, etc	2	CD36, COL3A1
Cell-To-Cell Signaling and Interaction	4.95E-10–4.99E-03	30	CCL2, CD74, CXCL1, CXCL2, CXCL9, ICAM1, IL8, NRG1, STAT1, etc.	3	CD36, SERPINI1, GPC3
Hematological System Development and Function	4.95E-10–5.95E-03	36	CCL2, CCL20, CXCL9, CXCL10, IL8, IL1RN, TNFAIP3, etc	1	CD36
Immune Cell Trafficking	4.95E-10–5.73E-03	26	CCL2, CCL20, CTSS, CXCL6, CXCL9, CXCL10, MDK, NEDD9, etc.	1	CD36
Infection Mechanism	5.03E-10–3.66E-03	16	CCL2, CXCL9, CXCL10, DDX58, IFIT1, IL8, ISG20, MX1, RSAD2, STAT1, etc.	0	
Infectious Disease	5.03E-10–5.46E-03	26	APOBEC3G, CXCL9, CXCL10, DDX58, MT1X, STAT1, TNFAIP3, etc	2	CD36, HMGCS1
Reproductive System Disease	6.43E-10–1.37E-03	42	CCL2, CXCL1, CXCL2, IFIT1, IGFBP1, KLF4, MAP3K8, NEDD9, SPP1, etc.	1	RNF5
Cellular Movement	6.64E-10–5.91E-03	31	IGFBP1, IL8, KLF4, MDK, NEDD9, NRG1, RARRES1, SOD2, TNFAIP8, etc	2	CD36, RNF5

### Change of gene expression with interferon treatment

**Figure 4:**
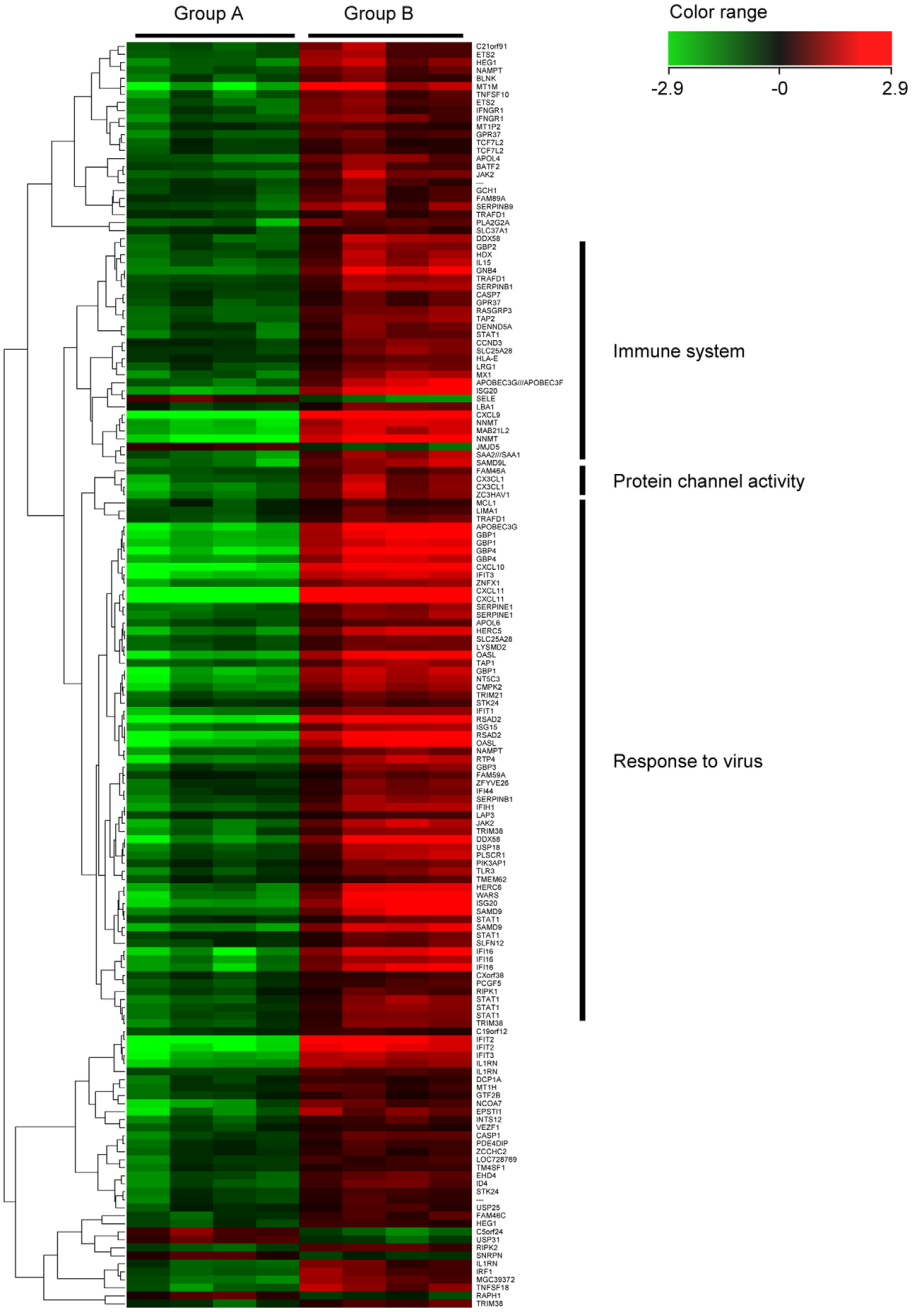
Hierarchical clustering analysis of 158 genes associated with IFN treatment. To analyze the effects of IFN in human hepatocytes, clustering analysis was performed between Group A (without IFN treatment; 4 columns on the left side) and Group B (with IFN treatment; 4 columns on the right side). 152 genes were up-regulated, and 6 genes were down-regulated following IFN treatment. Several well-known interferon-stimulated genes (ISGs), including *CXCL9*, *Mx1*, *ISG20* and *OASL,* were among the up-regulated genes.

To analyze the direct effects of IFN in human hepatocytes, we compared gene expression profiles between Group A (without IFN treatment) and Group B (with IFN treatment). Out of the 218 genes that remained significant after screening by Welch's t-tests and Benjamini-Hochberg correction for multiple testing, 158 had a greater than 3.0-fold change between groups. 152 of the 158 genes were up-regulated following IFN administration, and the other 6 were down-regulated. Cluster analysis of the 158 selected genes is shown in Figure 4. The top 35 up-regulated genes (>10.0-fold changes), which include many well-known ISGs (e.g., members of the *CXCL* and *IFI* families), and the 6 down-regulated genes are listed in Tables 4 and 5, respectively.

**Table 4:** The top 35 genes up-regulated with IFN treatment.

Probe set	Unigene code	Gene symbol	Fold change	P values
211122_s_at	Hs.632592	CXCL11	482.47	1.30E-06
203915_at	Hs.77367	CXCL9	216.26	1.35E-07
242625_at	Hs.17518	RSAD2	101.24	1.26E-05
202237_at	Hs.503911	NNMT	86.80	6.52E-06
217502_at	Hs.437609	IFIT2	75.05	1.73E-06
204533_at	Hs.632586	CXCL10	67.43	2.72E-07
217546_at	Hs.647370	MT1M	46.69	1.12E-04
235175_at	Hs.409925	GBP4	44.94	1.03E-06
204205_at	Hs.660143	APOBEC3G	43.39	5.55E-06
204747_at	Hs.714337	IFIT3	32.73	1.17E-06
218943_s_at	Hs.190622	DDX58	32.19	1.44E-04
33304_at	Hs.459265	ISG20	31.97	3.94E-05
202269_x_at	Hs.62661	GBP1	31.73	5.30E-06
210797_s_at	Hs.118633	OASL	31.59	9.21E-06
200629_at	Hs.497599	WARS	29.65	2.28E-04
206332_s_at	Hs.380250	IFI16	26.37	3.80E-05
210302_s_at	Hs.584852	MAB21L2	24.31	5.31E-07
228531_at	Hs.65641	SAMD9	18.65	1.45E-05
223298_s_at	Hs.487933	NT5C3	17.48	6.65E-06
219863_at	Hs.26663	HERC5	17.02	2.29E-05
225710_at	Hs.173030	GNB4	16.98	3.30E-05
219684_at	Hs.43388	RTP4	16.38	2.55E-05
212657_s_at	Hs.81134	IL1RN	15.18	1.33E-06
219352_at	Hs.529317	HERC6	14.86	1.31E-04
226702_at	Hs.7155	CMPK2	12.50	1.86E-05
205842_s_at	Hs.656213	JAK2	12.49	6.16E-05
230036_at	Hs.489118	SAMD9L	11.98	7.84E-05
214995_s_at	Hs.660143	APOBEC3F///APOBEC3G	11.62	1.58E-04
823_at	Hs.531668	CX3CL1	11.15	1.07E-04
203153_at	Hs.20315	IFIT1	10.84	5.93E-06
225076_s_at	Hs.371794	ZNFX1	10.40	1.38E-06
213069_at	Hs.477420	HEG1	10.37	3.52E-05
205483_s_at	Hs.458485	ISG15	10.34	1.29E-05
235276_at	Hs.546467	EPSTI1	10.21	2.10E-04
219209_at	Hs.163173	IFIH1	10.05	4.06E-05

**Table 5:** The top 6 genes down-regulated with IFN treatment.

Probe set	Unigene code	Gene symbol	Fold change	P value
206211_at		SELE	5.83	6.11E-05
224875_at		C5orf24	5.46	1.11E-04
227256_at	Hs.183817	USP31	3.94	7.27E-05
220070_at	Hs.145717	JMJD5	3.87	5.04E-05
1552482_at	Hs.471162	RAPH1	3.31	1.73E-04
226587_at	Hs.592473	SNRPN	3.17	6.13E-05

### The effect of HCV infection on IFN response

**Figure 5:**
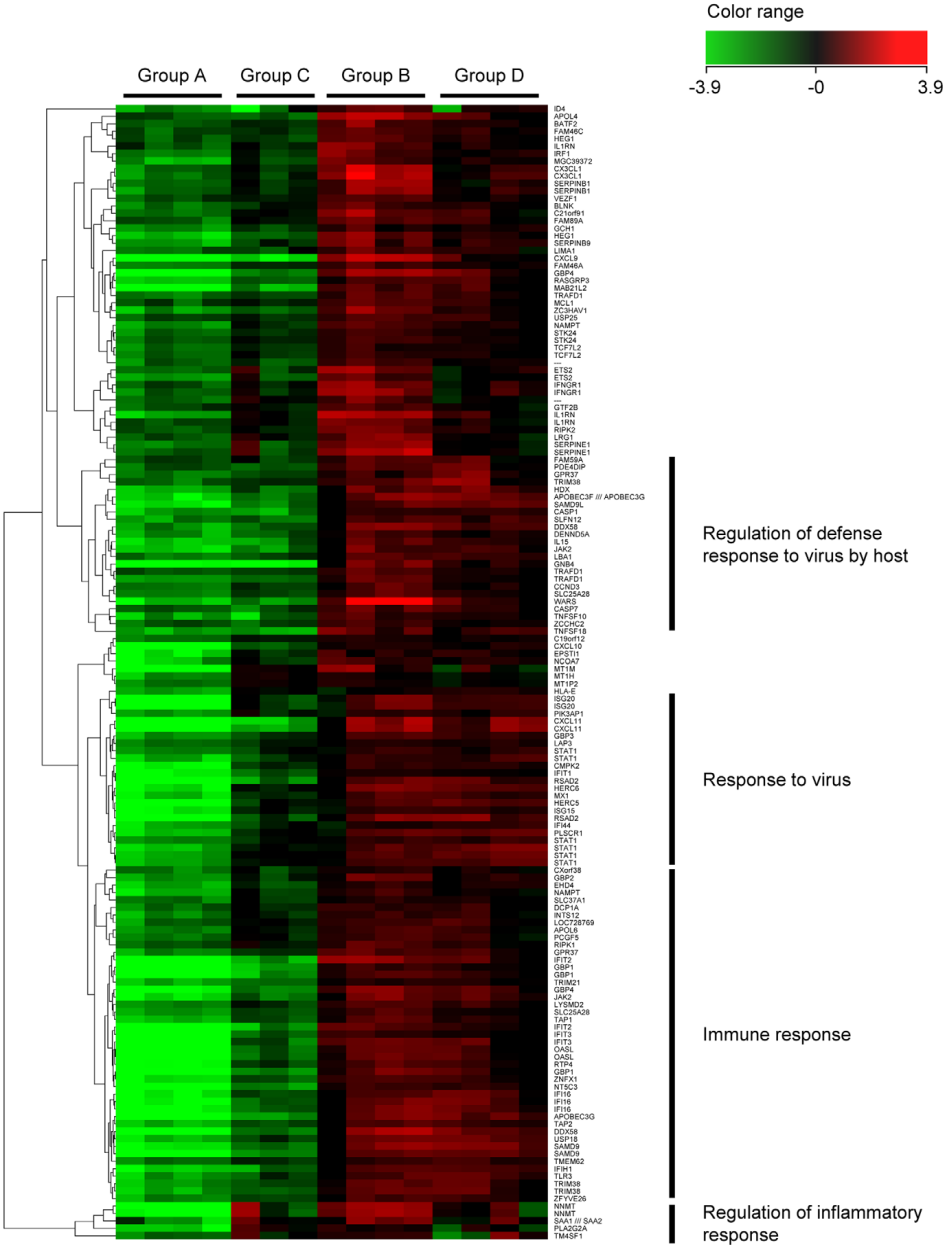
Hierarchical clustering analysis of 152 genes associated with IFN administration with or without HCV infection. To analyze the effect of HCV infection on IFN response, gene expression ratios between Groups A and B (gene expression changes by IFN without HCV infection) and those between Groups C and D (gene expression changes by IFN with HCV infection) were compared in the 152 IFN-induced genes. 69.7% of the selected genes showed reduced IFN responsiveness following HCV infection.

**Figure 6:**
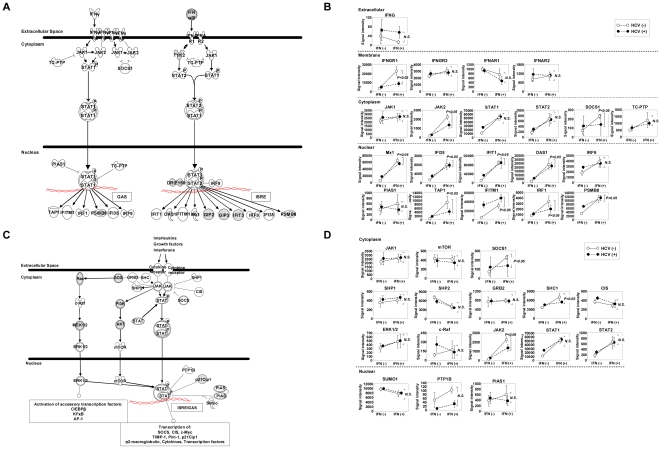
Changes in expression of genes in the IFN and JAK-STAT signaling pathways under HCV infection and/or IFN administration. A) An overview of the IFN signaling pathway consisting of 26 representative genes is shown. Genes illustrated as gray shapes were not included in this study. B) Relative expression levels of genes with/without HCV infection and/or IFN administration were plotted (closed dots: with HCV infection; open dots: without HCV infection) using microarray data. The slopes of the dashed and solid lines represent IFN responsiveness with and without HCV infection, respectively. In 21 of the 22 examined genes in the IFN signaling pathway, signal intensities increased and IFN responsiveness was repressed following HCV infection. Student's *t*-test was used for statistical analysis. C) An overview of the JAK-STAT signaling pathway consisting of 22 representative gene products is shown. Genes illustrated as gray shapes were not included in this study. D) Relative expression levels of genes with/without HCV infection and/or IFN administration were plotted using microarray data (closed dots: with HCV infection; open dots: without HCV infection). Signal intensities increased following HCV infection in 16 of 22 genes in the JAK-STAT signaling pathway, and IFN response was suppressed in 9 genes. Statistical analysis was performed using Student's *t*-test.

To analyze the effect of HCV infection on IFN response, we focused on the 152 genes that were up-regulated following IFN administration and compared gene expression ratios between Groups A and B (gene expression changes by IFN without HCV infection) and between Groups C and D (gene expression changes by IFN with HCV infection). In 69.7% (106/152) of the IFN-induced genes, IFN responsiveness was significantly reduced following HCV infection (Figure 5). The top 20 genes are shown in Table 6. Although viral titers differed among mice, we found no correlation between IFN responsiveness and HCV RNA titer. We performed pathway analysis to identify significant associations with canonical pathways, and the top 5 associated pathways are shown in Table 7. IFN responsiveness was significantly reduced following HCV infection in several canonical pathways, and the IFN signaling pathway, in particular, was strongly associated. To verify the effects of HCV infection and/or IFN treatment on gene expression, signal intensities of genes involved in the IFN and JAK-STAT signaling pathways were analyzed. As shown in Figure 6A, among 28 representative genes in the IFN signaling pathway, signal intensities of 22 genes could be analyzed through cDNA microarray analysis. In all genes except *IFNAR1*, expression was up-regulated following HCV infection, whereas IFN responsiveness was suppressed as a result of HCV infection (Figure 6B). 16 out of 22 genes in the JAK-STAT signal pathway could be analyzed via cDNA microarray analysis (Figure 6C), and 12 of the 16 genes were up-regulated following HCV infection, whereas IFN responsiveness was suppressed in 9 genes (Figure 6D).

**Table 6:** The top 20 genes in which IFN-induced up-regulation is inhibited following HCV infection.

Probe Set ID	Gene symbol	Fold change		P value
		HCV infection (-)	HCV infection (+)	
235175_at	GBP4	44.94	5.50	2.93E-07
231577_s_at	GBP1	24.60	4.89	6.15E-07
218943_s_at	DDX58	32.19	5.56	1.26E-05
226702_at	CMPK2	12.50	2.13	2.35E-05
225973_at	TAP2	7.07	2.84	3.31E-05
229450_at	IFIT3	20.66	2.74	6.37E-05
217739_s_at	NAMPT	5.91	1.95	6.51E-05
213797_at	RSAD2	69.70	4.50	7.01E-05
210797_s_at	OASL	31.59	3.53	1.02E-04
218508_at	DCP1A	3.56	1.65	1.36E-04
228531_at	SAMD9	18.65	5.87	1.45E-04
204804_at	TRIM21	5.37	2.69	1.47E-04
219209_at	IFIH1	10.05	4.69	1.67E-04
219684_at	RTP4	16.38	2.55	1.98E-04
239186_at	MGC39372	7.61	2.57	2.27E-04
219211_at	USP18	8.72	3.74	2.40E-04
225076_s_at	ZNFX1	10.40	2.91	2.91E-04
204698_at	ISG20	31.29	3.33	3.00E-04
223192_at	SLC25A28	5.05	2.20	3.25E-04
228439_at	BATF2	4.59	1.83	3.28E-04

**Table 7:** The top 5 canonical pathways associated with the 106 genes in which IFN response is suppressed by HCV infection.

Category	P value	Ratio	Associated genes
Interferon Signaling	4.41E-11	7/30 genes	IFIT1, IFIT3, IFNGR1, IRF1, MX1, STAT1, TAP1
Type I Diabetes Mellitus Signaling	1.08E-04	5/119 genes	HLA-E, IFNGR1, IRF1, RIPK1, STAT1
Antigen Presentation Pathway	5.29E-04	3/39 genes	HLA-E, TAP1, TAP2
Primary Immunodeficiency Signaling	1.64E-03	3/63 genes	BLNK, TAP1, TAP2
Activation of IRF by Cytosolic Pattern Recognition Receptors	2.95E-03	3/73 genes	ISG15, RIPK1, STAT1

On the other hand, only 33 genes (21.7%), including several ISGs, such as *GBP1*, *GBP4* and *IFIT3*, remained responsive to IFN in the presence of HCV and were expressed more than 3.0-fold higher in Group D compared to Group C (Table 8). Pathway analysis indicated that these 33 genes were significantly associated with Antimicrobial Response and Inflammatory Response (P = 5.22×10^−10^ ∼ 1.95×10^−2^). Changes in mRNA expression for 29 down-regulated genes, including *ISG20*, *WARS*, *Mx1*, *CXCL10*, *IFNGR1* and *IFITM1* were verified by real time PCR (data not shown).

**Table 8:** The 33 genes that remained more than 3-fold up-regulated following IFN treatment in HCV-infected mice.

ID	Symbol	Location	Type(s)
214995_s_at	APOBEC3F	unknown	enzyme
204205_at	APOBEC3G	Nucleus	enzyme
206011_at	CASP1	Cytoplasm	peptidase
204533_at	CXCL10	Extracellular Space	cytokine
210163_at	CXCL11	Extracellular Space	cytokine
203915_at	CXCL9	Extracellular Space	cytokine
218943_s_at	DDX58	Cytoplasm	enzyme
231577_s_at	GBP1 (includes EG:2633)	Cytoplasm	enzyme
235175_at	GBP4 (includes EG:115361)	Cytoplasm	enzyme
225710_at	GNB4	Plasma Membrane	enzyme
1553646_at	HDX	unknown	other
213069_at	HEG1	unknown	other
206332_s_at	IFI16	Nucleus	transcription regulator
219209_at	IFIH1	Nucleus	enzyme
217502_at	IFIT2	unknown	other
204747_at	IFIT3	Cytoplasm	other
205992_s_at	IL15	Extracellular Space	cytokine
204698_at	ISG20	Nucleus	enzyme
205841_at	JAK2	Cytoplasm	kinase
210302_s_at	MAB21L2	unknown	other
223298_s_at	NT5C3	Cytoplasm	phosphatase
205660_at	OASL	unknown	enzyme
205801_s_at	RASGRP3	Cytoplasm	other
242625_at	RSAD2	unknown	enzyme
228531_at	SAMD9	unknown	other
230036_at	SAMD9L	unknown	other
219885_at	SLFN12	Nucleus	enzyme
206271_at	TLR3	Plasma Membrane	transmembrane receptor
214329_x_at	TNFSF10	Extracellular Space	cytokine
221371_at	TNFSF18	Extracellular Space	cytokine
203610_s_at	TRIM38	unknown	other
219211_at	USP18	Cytoplasm	peptidase
200629_at	WARS	Cytoplasm	enzyme

## Discussion

We previously developed a human hepatocyte chimeric mouse model that can be chronically infected with hepatitis B and C viruses [29]–[31]. This mouse model has enabled us to analyze the effect of viral infection and the response to medication under immunodeficient conditions. Microarray analyses using the human hepatocyte chimeric mouse model with HCV infection have recently been reported, and HCV infection was found to affect expression of genes related to innate antiviral immune response, lipid metabolism and apoptosis via ER stress [34], [35]. Whereas these reports were concerned especially with host specific responses to HCV infection, no studies addressing viral modulation of the IFN response have been reported, even though such studies might be important for understanding viral evasion mechanisms in response to IFN therapy and for improving therapy effectiveness for chronic hepatitis C. Therefore, in this study we performed cDNA microarray analysis using a human hepatocyte chimeric mouse model and obtained gene expression profiles to investigate direct influences of HCV infection on IFN responses in human hepatocytes.

First, we evaluated host response to HCV infection in human hepatocytes by comparing profiles between groups A (without HCV infection) and C (with HCV infection). 181 genes were significantly up- or down-regulated following HCV infection. Canonical pathway analysis revealed that genes involved in IFN signaling were the most strongly up-regulated following HCV infection (Figure 3). These findings are mostly consistent with previous studies [36], [37]. On the other hand, while no genes involved in lipid metabolism showed any significant induction by HCV infection in this study, Walters et al. reported that HCV-infected chimeric mice exhibited host-specific induction in the expression of lipid metabolism genes [35]. However, we used hepatocytes from a single donor, whereas Walters et al. used hepatocytes from multiple donors, so our results are not necessarily inconsistent with their findings that HCV infection causes induction of lipid metabolism genes in a host-specific manner.

Although several cDNA microarray analyses have also been performed using human liver tissues obtained after hepatic resection, the largest difference between human and chimeric mouse livers is the presence or absence of human lymphocytes. According to the previous report using human liver tissues, genes involved in the innate immune response, as well as cell cycle, growth and communication, were up-regulated by HCV infection [38]. In the present study using SCID-derived mice, genes involved in immune response (e.g. *OAS2*, *Mx1*, *IFI27* and *IFI44L*), cell cycle and growth (e.g. *HERC5*) and cell communication (e.g. *HLA-B*) were similarly up-regulated by HCV infection. However, *Apolipoprotein L*, *Cold autoinflammatory syndrome 1*, *CD97 antigen*, and *HLA-DQ*, which are mainly expressed in lymphocytes, were not observed to be up-regulated by HCV infection in the chimeric mice. These results demonstrate that the chimeric mouse model accurately reflects intracellular responses to HCV infection without the lymphocytic immune response.

To verify the microarray results, expression data were compared with previously published microarray data on the GEO website (http://www.ncbi.nlm.nih.gov/geo/). Previously published microarray data showed up-regulation of *IGFBP7*, *IFI27*, *HLA-B*, and *CD74* in HCV-infected liver tissues compared to non-infected liver tissues (fold changes were 2.1, 2.2, 2.1 and 2.3, respectively) [39]. Likewise, we found that *IFI27*, *HLA-B*, and *CD74* were up-regulated following HCV infection (fold changes were 3.6, 3.3, and 6.6, respectively). These three genes are associated with MHC class I activity, suggesting that intra-cellular immunity in human hepatocytes was activated following HCV infection both in human subjects and in chimeric mouse livers. Metallothionein 1G (*MT1G*) expression was also found to be up-regulated by HCV infection in both the current and published studies [39], [40]. Although metallothionein isoforms are associated with collagen deposition [41], members of the metallothionein family may be up-regulated and induce liver fibrosis in response to HCV infection.

In this study, genes associated with Organismal Injury and Abnormalities were found to be up-regulated in response HCV infection (Table 3), and some genes in this category, such as *CXCL9*, *CXCL10* and *IFIT3*, maintained high IFN responsiveness under HCV infection (Table 8). These results suggest that protective responses to fibrosis or hepatic injury were activated at the start of HCV infection and remained activated until complete eradication of HCV from hepatocytes was achieved.

Secondly, we compared gene expression profiles between groups A (without IFN treatment) and B (with IFN treatment) to evaluate IFN response without HCV infection. IFN-α stimulates the intracellular IFN-signaling cascade after binding to the IFN-α receptor and mediates the transcriptional activation of IFN-stimulated genes [42]–[47]. More than 3.0-fold up-regulation was observed 6hrs after IFN treatment in 152 genes. Known ISGs such as those in the *CXCL* family (*CXCL9*, *CXCL10* and *CXCL11*), the *IFIT* family (*IFIT2* and *IFIT3*) and the *APOBEC* family (*APOBEC3G*) were included among the top 20 genes up-regulated following IFN treatment (Table 4). The *APOBEC* family is well known to have anti-viral effects by inducing genomic hypermutation in human immunodeficiency virus and hepatitis B virus [48]–[57]. *APOBEC3G* expression has been reported to be elevated in patients infected with HCV [58], although it is not clear whether *APOBEC3G* can block HCV replication. On the other hand, *CXCL9* and *IFIT3* were reported to relate to liver fibrosis in chronic hepatitis C patients. Serum *CXCL9* concentrations correlated with the levels of fibrosis in chronic hepatitis C patients, and *CXCL9* has been shown to exert anti-fibrotic effects *in vitro* and *in vivo* [59]. *IFIT3* expression is also reportedly up-regulated in the transition from mild to moderate fibrosis [60]. The results of this study suggest that IFN treatment might lead not only to HCV eradication but also help to prevent and repair liver fibrosis by inducing these key molecules.

We focused on the 152 genes up-regulated (> 3.0 fold) as a result of IFN administration and evaluated the effect of HCV infection on IFN response among these genes. As shown in Table 8, although several ISGs still showed high response to IFN treatment in the presence of HCV infection, 7 genes in the IFN Signaling pathway became unresponsive (Table 6). Reduction in IFN responsiveness was also observed for *STAT1* (4.27 fold in the absence of HCV to 2.29 fold in the presence of HCV, P = 4.04×10^−4^), as well as 5 of 7 genes downstream of *STAT1* (*IFIT1*, *IFIT3*, *IRF1*, *MX1*, and *TAP1*). As shown in Figure 3, IFN signaling was activated in the presence of HCV, and the expression of *STAT1* was more than 3.0 fold up-regulated by HCV infection (data not shown). *STAT1* expression was highest in mice with both HCV infection and IFN treatment, but downstream genes such as *MX1*, *IFIT1* and *IFIT3* showed reduced IFN response. Sarasin-Filipowicz et al. reported that IFN-induced *STAT1* phosphorylation was stronger in rapid responders than in non-rapid responders [61]. Reduced induction of genes downstream of *STAT1* by IFN under HCV infection might reflect reduced phosphorylation of *STAT1*, although we did not quantify *STAT1* phosphorylation in this study.

Recently, an *IL-28B* genetic polymorphism strongly associated with response to IFN-α plus ribavirin combination therapy [12], as well as with hepatic ISG expression [62], was identified. Further studies using chimeric mice transplanted with hepatocytes carrying different genotypes of candidate genes such as *IL-28B* will be important in order to elucidate possible mechanisms underlying host-specific responses.

In conclusion, we performed cDNA microarray analysis using HCV-infected human hepatocyte chimeric mice, which allowed us to analyze the direct effects of IFN treatment and HCV infection without the confounding effects of the lymphocytic immunological response. These results might provide molecular insights into possible mechanisms used by HCV to evade IFN-induced immune responses, as well as suggest novel therapeutic targets and a potential new indication for interferon therapy. Further analysis of the genes identified in our study would be worthwhile in order to improve efficacy of the therapy for chronic hepatitis C.
